# Omics Approaches to Study Perilipins and Their Significant Biological Role in Cardiometabolic Disorders

**DOI:** 10.3390/ijms26020557

**Published:** 2025-01-10

**Authors:** Erica Gianazza, Giulia G. Papaianni, Lisa Brocca, Cristina Banfi, Alice Mallia

**Affiliations:** Unit of Functional Proteomics, Metabolomics and Network Analysis, Centro Cardiologico Monzino IRCCS, 20138 Milan, Italy; erica.gianazza@cardiologicomonzino.it (E.G.); giulia.papaianni@cardiologicomonzino.it (G.G.P.); lisa.brocca@cardiologicomonzino.it (L.B.); alice.mallia@cardiologicomonzino.it (A.M.)

**Keywords:** lipid droplets, perilipin, proteomics, omics approaches, cardiovascular diseases, cardiometabolic disorders

## Abstract

Lipid droplets (LDs), highly dynamic cellular organelles specialized in lipid storage and maintenance of lipid homeostasis, contain several proteins on their surface, among which the perilipin (Plin) family stands out as the most abundant group of LD-binding proteins. They play a pivotal role in influencing the behavior and functionality of LDs, regulating lipase activity, and preserving a balance between lipid synthesis and degradation, which is crucial in the development of obesity and abnormal accumulation of fat in non-adipose tissues, causing negative adverse biological effects, such as insulin resistance, mitochondrial dysfunction, and inflammation. The expression levels of Plins are often associated with various diseases, such as hepatic steatosis and atherosclerotic plaque formation. Thus, it becomes of interest to investigate the Plin roles by using appropriate “omics” approaches that may provide additional insight into the mechanisms through which these proteins contribute to cellular and tissue homeostasis. This review is intended to give an overview of the most significant omics studies focused on the characterization of Plin proteins and the identification of their potential targets involved in the development and progression of cardiovascular and cardiometabolic complications, as well as their interactors that could be useful for more efficient therapeutic and preventive approaches for patients.

## 1. Lipid Droplet Biogenesis and Their Functions

Lipid droplets (LDs) are ubiquitous organelles consisting of a neutral central lipid core (e.g., triacylglycerol (TAG) and sterol esters), surrounded by a phospholipid monolayer, and containing both LD-related and non-specific proteins on their surface [1]. They are highly dynamic organelles that continuously change between periods of expansion and degradation through lipase-catalyzed hydrolysis or a selective autophagic mechanism called lipophagy.

**Figure 1:**
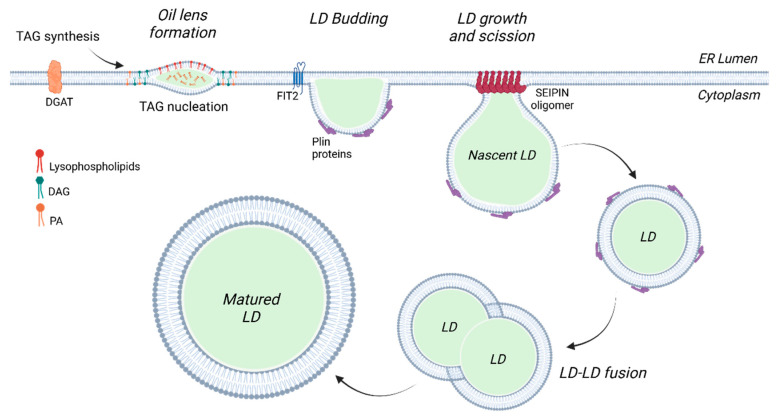
Multiple steps in lipid droplet (LD) formation and expansion. LD biogenesis occurs in the endoplasmic reticulum (ER), and several enzymes, including diacylglycerol acyltransferases (DGATs), catalyze the synthesis of neutral lipids between the leaflets of the ER bilayer. An increase in neutral lipid concentration promotes the formation of an oil lens in a process of demixing, after which follows LD budding as a result of the expansion of the neutral lipid lens. LD growth and maturation involve the transformation of a neutral lipid lens into a spherical LD that undergoes release from the ER and eventually droplet–droplet fusion. DAG, diacylglycerol; FIT2, fat storage-inducing transmembrane 2; PA, phosphatidic acid; Plin, perilipin; TAG, triacylglycerol.

The biogenesis of LDs involves multiple steps and occurs within the membrane of the endoplasmic reticulum (ER), where the enzymes responsible for neutral lipid formation are located (Figure 1). Several mechanisms have been proposed regarding the formation of LDs, but it is still unclear today whether they form randomly within the ER or at specialized subdomains of the ER. As the concentration of neutral lipids increases, they begin to coalesce, creating an oil lens during a demixing process. This results in the generation of early LD intermediates, which are typically small and short-lived [2]. Therefore, the budding of LDs from the ER membrane results from the expansion of the neutral lipid lens. It has also been observed that both the ER membrane bilayer phospholipid composition and surface tension affect LD formation [3]. ER membrane phospholipids have distinct LD budding abilities, and specific phospholipids (e.g., lysophospholipids) can decrease tension, favoring the formation of small LDs. Reducing tension promotes neutral lipid escape from the ER membrane bilayer and thus, LD budding.

However, the spatial and temporal organization of LDs, as well as the proteins and lipids involved in this process, are yet to be fully defined [4]. Studies suggest that fat storage-inducing transmembrane (FIT) proteins, which are a conserved family of integral ER membrane proteins, favor LD budding [5]. Furthermore, members of the perilipin (Plin) family have been identified as playing a key role in the formation of LDs and their maintenance [6,7].

Seipin is another highly conserved ER membrane protein that plays a crucial role in assembling and maintaining nascent LDs [8]. Seipin regulates LD biogenesis and morphology and incorporates protein and lipid cargo into growing LDs in human cells thanks to its contribution to stabilizing ER-LD contacts that provide continuity between the two organelles [9,10]. In addition, the presence of a high number of intranuclear droplets in the seipin-null *S. cerevisiae* strain has been described, suggesting a key role of seipin in droplet budding directionally toward the cytoplasm [9].

After budding, LDs grow through droplet–droplet fusion, local synthesis of TAG on the LD surface, or transfer of TAG to LDs via membrane bridges from the ER [2].

LDs connect with the ER through membrane bridges, as well as with a variety of other cellular organelles, including the Golgi, mitochondria, lysosomes, peroxisomes, and cytoskeleton, through heterotypic membrane contact sites (MCSs). These associations are essential for the biogenesis and expansion of LDs, autophagy, and transfer of cellular components [11]. In particular, LD-ER contact sites are crucial for LD formation, growth, and budding [12], as well as for removing damaged and unfolded proteins from the ER. Toxic lipids or proteins present in LDs can also be transported to lysosomes via LD–lysosome contact sites [13]. Instead, MCSs between LDs and the cytoskeleton facilitate the movement and motility of LDs [13]. In addition, the MCSs between LDs and mitochondria are important for transferring fatty acids (FAs) produced from neutral lipids in LDs to mitochondria, which are oxidized for energy production [11]. These interaction sites are primarily localized in energy-intensive tissues such as the heart, brown adipose tissue (BAT), liver, and skeletal muscle. In fat-oxidizing tissues, a subpopulation of mitochondria known as peridroplet mitochondria (PDM) is closely associated with large LDs. These PDM have a unique composition and function that regulate the expansion of LDs and the synthesis of triacylglyceride, promoting lipid storage [14].

LDs are involved in intracellular lipid metabolism, playing several roles in various cell types, including lipid storage, intracellular membrane trafficking, and protein maturation and degradation [15].

LDs regulate the levels of potentially toxic lipids in cells, reducing lipotoxicity and oxidative stress [2,16]. LDs play a protective role against ER stress, which occurs when there are alterations in ER protein folding capacity, lipid composition, and calcium uptake. The accumulation of misfolded proteins or toxic lipids can lead to ER stress. Additionally, any alteration in the biogenesis or storage capacity of LDs can trigger an upregulation of the unfolded protein response, a cellular mechanism designed to restore ER homeostasis [17].

LDs also help protect against the accumulation of acylcarnitines and mitigate lipotoxic damage to mitochondria during autophagy [18]. They sequester the excess FAs released during the autophagic degradation of membranous organelles and transfer them to the ER. There, diacylglycerol acyltransferase 1 (DGAT1) catalyzes their conversion to TAGs, which are stored in LDs derived from the ER. TAGs conserved in LDs are also utilized to produce FAs, which are then converted into acylcarnitine and transported into mitochondria for ATP production via β-oxidation [19]. Elevated levels of acylcarnitines are harmful to mitochondrial function, emphasizing the important interplay between LDs, acylcarnitines, and cellular dysfunction. This connection is especially relevant in human cardiac diseases [2], where damage associated with acylcarnitine plays a pivotal role in the lipotoxicity observed in the cardiac tissues [20].

LDs are primarily defined by their associated proteins. The size and protein composition of these droplets can vary significantly among different tissue and cell types. A diverse range of proteins plays a pivotal role in influencing the behavior and functionality of LDs. These proteins are categorized into two classes: Class I proteins, which are initially associated with the ER membrane and have the potential to partition into LDs, and Class II proteins, which are recruited from the cytosol to the surface of the LDs. Class II proteins interact with the phospholipid monolayer through various mechanisms, including amphipathic helices or lipid-anchors, and protein–protein interactions [21].

## 2. Perilipin Proteins: Structure and Functions

Proteomics studies have identified hundreds of proteins associated with LDs, each fulfilling various functions [22,23,24]. Among these, the Plin family stands out as the most abundant group of LD-binding proteins, comprising five distinct isoforms called Plin1, Plin2, Plin3, Plin4, and Plin5. Collectively known as the PAT family, this group takes its name from the initials of its first three members: perilipin 1 (Plin1), Adipophilin (also known as perilipin 2, Plin2), and Tip47 (also known as perilipin 3, Plin3) [25,26].

This group of proteins was identified in 1991 by Greenberg et al. as phosphoproteins associated with LDs in cultured adipocytes. This discovery not only highlighted the specificity of these proteins to adipocytes but also suggested their vital role in the lipid storage functions of these cells [27].

**Figure 2:**
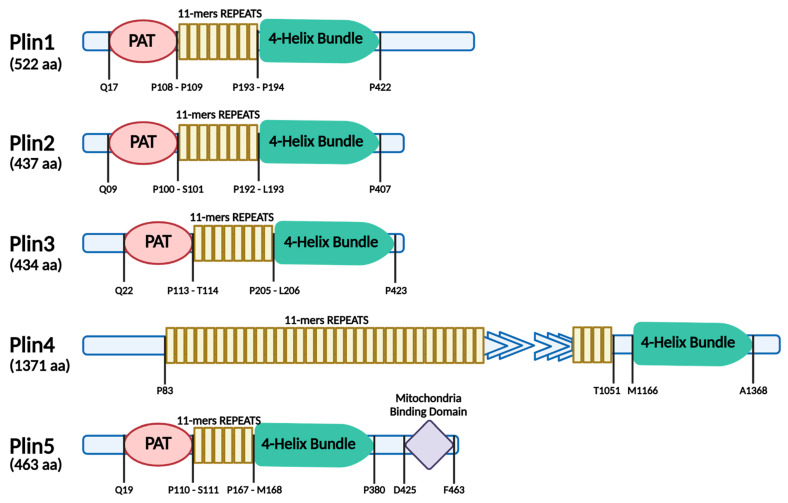
Protein structure of the different Plin isoforms. The Plin family includes five isoforms (Plin1 to Plin5) that are characterized by an 11-mer repeat motif and a conserved PAT domain, with the exception of Plin4. The C-terminal 4-helix bundle structure is present in all Plin proteins [33].

Perilipins belong to Class II proteins that target LDs directly from the cytosol. All members of the Plin family of proteins contain a conserved N-terminal amino acid sequence characterized by 11-mer repeats, which is predicted to adopt an amphipathic helical structure [28]. Furthermore, these proteins exhibit considerable similarities in other regions as well (Figure 2). Plins bind to LDs through hydrophobic helices that integrate into the droplet surface. They are transported from the Golgi apparatus to the ER via the ADP-ribosylation factor 1-coat protein complex I (Arf-COPI), which is essential for regulating the formation, structure, and expansion of LDs [29].

The five Plin proteins are numbered based on their discovery. A comparison of the sequences of all members reveals that Plin2 and Plin3 have the highest overall similarity, and both are ubiquitously expressed [30]. Plin5 has a protein structure similar to Plin2 and Plin3 in its entirety; however, it is expressed mainly in tissues in which FAs released during lipolysis are delivered to mitochondria for oxidation. Indeed, this protein contains a peculiar domain that allows it to interact with mitochondria [31]. Plin1’s structure is similar to that of Plin2, 3, and 5 at the N-terminal region but differs at the carboxyl terminus [32]. Lastly, Plin4 has the most divergent protein structure compared to the other members of Plins, presenting an extended 11-mer repeated region [28]. The common and unique characteristics of Plin proteins are summarized in Table 1.

It has been established that Plins preferentially localize specific types of intracellular lipid storage droplets. In particular, Plin1 and Plin5 target TAG-specific droplets, while the shorter isoforms of Plin1 (i.e., Plin1c) and Plin4 associate with cholesteryl ester-specific droplets [34]. Instead, Plin2 and Plin3 do not have an exclusive association and interact both with TAG-specific and cholesteryl ester-specific LDs. Many cells present small LDs characterized by the presence of Plin2 and Plin3 on their surface, while cells specialized in fat storage show larger LDs that express Plin1, Plin4, and/or Plin5 [35].

Future studies aimed at elucidating the high-resolution structures of full-length Plins in their lipid-bound forms will be crucial for enhancing our understanding of the molecular mechanisms that govern their targeting to LDs and their regulatory roles in lipolysis [35].

**Figure 3:**
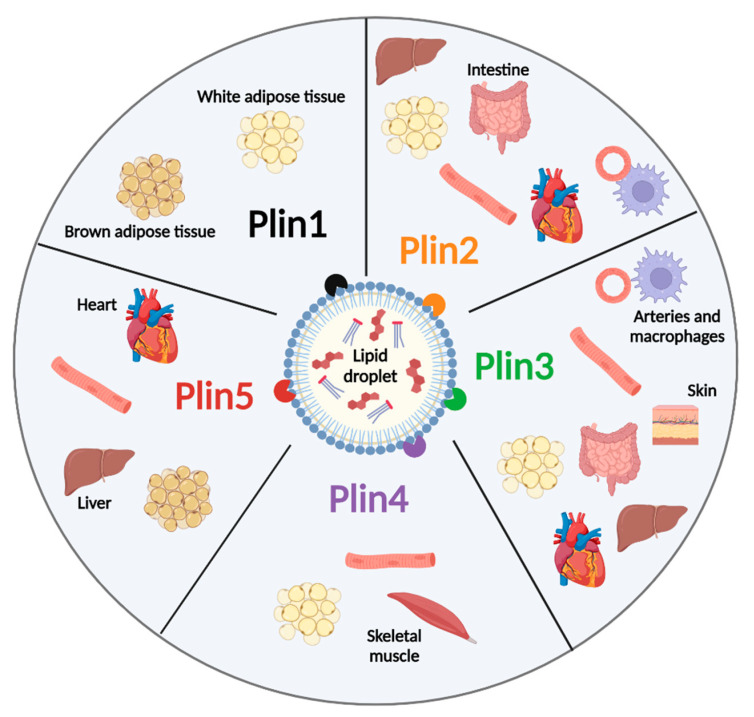
Distribution of Plin proteins in different organs and tissues.

While Plins 2 and 3 are ubiquitously expressed, Plins 1, 4, and 5 have a tissue-specific expression. In particular, Plin1 is abundantly expressed in white and brown adipose tissues, where it is also involved in LD–mitochondria contact site formation through the interaction with mitofusin 2 [36] and, to a lesser extent, in steroidogenic cells [35]. Plin2 is ubiquitously expressed but is relatively more abundant in the liver. Plin4 is mostly expressed in the white adipose tissue (WAT), whereas Plin5 is highly present in oxidative tissues, including the cardiac muscle, liver, skeletal muscle, and BAT (Figure 3).

Perilipins are essential in regulating lipase activity by inhibiting the hydrolysis of LDs and sustaining a balance between lipid synthesis and degradation [37]. Due to their critical role in lipid metabolism, Plins have been identified as promising targets for treating lipid-related diseases. They help maintain the equilibrium of various FA stores, which is crucial in the development of conditions such as obesity and the abnormal accumulation of fat in non-adipose tissues, including hepatic steatosis and atherosclerotic plaque formation [37,38,39].

Plin1, Plin2, and Plin5 play a crucial role in regulating adipose lipolysis, whether in basal or fed conditions [35]. They achieve this through distinct mechanisms that limit the access of lipases and co-factors to lipid substrates stored within LDs, thereby promoting TAG storage under basal conditions. In the fed state, the insulin-mediated reduction in protein kinase A (PKA) activity further supports this function.

Since the proteome of LDs is integral to their biological functions, influencing processes such as lipid storage and metabolism, and plays a significant role in various aspects of their biology, Plins represent the most extensively studied proteins that are associated with the surface of LDs [40]. LD accumulation in cells and tissues acts as a protective mechanism to prevent lipotoxicity caused by lipid overload. Nevertheless, an excessive accumulation of LDs is frequently associated with negative adverse biological effects, such as insulin resistance, mitochondrial dysfunction, and inflammation [40,41]. As a result, the expression levels of Plins are often associated with a variety of diseases. Indeed, Plins are involved in the development of obesity [42], diabetes [43], hepatic disorders [39], atherosclerosis and other heart diseases [44,45,46,47], and cancer, which are all caused by altered lipid metabolism [38]. Plins are abundantly expressed in macrophages and foam cells, as well as in cardiac tissue, and their altered expression causes cardiac LD accumulation and inflammation, increasing the risk of unstable atherosclerotic plaques, hypertension, myocardial ischemia, and hypertrophic cardiomyopathy [48].

Changes in the abundance of Plins generate an excessive retention of lipids in LDs, favoring abnormal lipid metabolism and obesity, which are defining characteristics of the development of cancer and other comorbidities [37,49]. The close interplay between Plins and cancer is extensively documented in several types of carcinoma, including breast [50,51], lung [52], kidney [53,54], melanoma [55], and liver [56], in which Plins are associated with tumorigenesis and are often recognized as poor prognosis indicators. Thus, in the context of cancers, Plins are considered potential biomarkers for an early diagnosis and prognosis and targets for a pharmacological intervention.

Therefore, research interest has focused on the characterization of Plin proteins, the identification of the mechanisms through which these proteins contribute to cellular and tissue homeostasis, and the development of therapeutic approaches to regulate their functions in specific pathological conditions [40].

Plin proteins are differently involved in several human diseases associated with impaired lipid metabolism, including cardiovascular diseases (CVDs). Lipid homeostasis in cardiomyocytes is based on a critical balance between FA uptake and consumption. Myocardial triglycerides (TGs) are stored in cytosolic LDs, and they are physiologically low, but in pathological conditions, they can significantly increase. Myocardial dysfunction is frequently associated with an abnormal increase in cardiac LDs [57].

Since Plins are the most abundant LD proteins, they are involved in myocardial lipid dynamics, and they attracted interest because of their critical role in cardiac function. Numerous studies demonstrated the important connection between Plins and CVDs, highlighting that these proteins control the accessibility of the TG pool and determine the hydrolysis of LDs in cardiac tissue. Moreover, the interplay between Plins is critical to maintaining the correct lipid metabolism in the heart.

Plins, with the exception of Plin4, are expressed in the heart at different levels and amounts, but their role remains controversial both in mice and humans, generating conflicting points of view [44]. Some variants have been reported to have a protective effect against CVDs by reducing pro-inflammatory macrophages that form unstable atheroma plaques (e.g., [58]), but at the same time, evidence of atherosclerosis promotion has also been reported following excessive lipid accumulation and foam cell generation (e.g., [57]). A comparative table of the Plins in different atherosclerosis-related conditions is reported (Table 2).

Animal models have given important insights into how Plins contribute to CVDs, highlighting both conserved functions and species-specific differences. In rodents, Plin5 is crucial in protecting the heart by sequestering FAs in LDs, reducing oxidative stress, and protecting mitochondrial function. In mice, Plin5 deficiency is linked to an increase in reactive oxygen species (ROS) production and impaired cardiac functions, highlighting its importance in regulating lipid metabolism within the heart [67,70]. Similarly, Plin isoforms show differential regulation under physiological and pathological conditions, such as during pregnancy-induced cardiac hypertrophy in rats, where increased expression of Plin1, Plin2, and Plin5 is observed [71].

Despite these similarities, notable interspecies variations exist. For example, Plin5’s mitochondrial tethering and its role in LD dynamics are more pronounced in rodent hearts compared to larger animals, such as pigs and rabbits. Furthermore, while lipid metabolism mechanisms in pigs and rabbits offer physiological parallels to humans, their use in Plin-specific cardiovascular studies remains limited, necessitating further investigation to bridge translational gaps [72]. This comparative analysis underscores the necessity of studying the role of Plins in diverse animal models to fully elucidate protein functions in cardiovascular health and disease.

This review aims to provide information on the structure and functions of these proteins in regulating the physiological processes in myocardial and vascular cells and give an overview of the most significant omics studies focused on their potential targets involved in the development and progression of cardiometabolic complications.

## 3. Perilipin 1

### 3.1. Structure and Functions of Plin1

Perilipin 1 is primarily located in mature adipocytes, where it regulates TAG storage within LDs [44]. It is recognized as the most heavily phosphorylated protein associated with LDs in stimulated adipocytes, and its mechanisms for controlling lipolysis are affected by PKA-mediated phosphorylation [35,37].

In humans, Plin1 is a 522-amino acid sequence protein that includes a region of 11-mer repeats predicted to form amphipathic helices, which is conserved in all proteins of the Plin family [35]. In addition, Plin1 has a hydrophobic domain within the N-terminal region known as the PAT domain, three sequences of hydrophobic amino acids with central proline residues, which are involved in the targeting and anchoring of Plin1 to LDs [73], and five consensus sites for the phosphorylation of serine residues by PKA: Ser81 and Ser277 in the N-terminal end and Ser436, Ser497, and Ser522 in the C-terminal end [44]. This phosphorylation is known to quickly initiate lipolysis.

Under basal conditions, Plin1 associates with abhydrolase domain containing 5 (ABHD5) through a carboxyl-terminal sequence, forming a barrier on the surface of LDs. This interaction prevents ABHD5 from engaging with adipose triglyceride lipase (ATGL) [74] and limits access to cytosolic lipases. Upon activation of PKA, ABHD5 is released from the Plin1 scaffold, enabling it to interact with phosphorylated ATGL. This interaction leads to a substantial increase in hydrolytic activity against diacylglycerols (DAGs), cholesterol esters (CEs), and TAGs. Hormone-sensitive lipase (HSL), which is also phosphorylated by PKA, translocates from the cytoplasm to the surfaces of LDs. This process facilitates the hydrolysis of DAG into monoacylglycerol (MAG) and FAs. Following this, MAG lipase, whose activity and localization to LDs are likely regulated by Plin1, converts MAG into free FAs and glycerol.

The aquaglyceroporin AQP7, which is a membrane protein known for transporting glycerol in adipocytes [75], has been identified as an interaction partner of Plin1 in human primary adipocytes, and its mobility is regulated by PKA-mediated phosphorylation [76]. Indeed, AQP7 plays a crucial role in fat accumulation, and its presence around LDs increases with insulin treatment. Under lipogenic conditions, Plin1 interacts with AQP7 following the phosphorylation of AQP7’s N-terminus by PKA. In contrast, the formation of this complex diminishes during lipolytic conditions. When Plin1 is absent or dysregulated, AQP7 can translocate to the plasma membrane, facilitating the efflux of glycerol. This process is a hallmark of lipolysis.

Several variants have been identified in *PLIN1*, the gene encoding Plin1, that emphasize its crucial involvement in human metabolism. It has been shown that these variants are implicated in acute coronary syndrome [77] and several familial partial lipodystrophies [78,79]. Moreover, individuals with Plin1 protein-truncating variants had a favorable metabolic profile that may protect against CVDs, reducing the risk of myocardial infarction and hypertension [80].

### 3.2. Role of Plin1 in Atherosclerosis and CVDs

Plin1 is not basally expressed in macrophages, but its upregulation occurs during foam cell production. In particular, Plin1 coats larger LDs in comparison to Plin2, which is expressed only in smaller LDs, as recently reported [35,81].

Several studies confirmed the contribution of Plin1 to atherosclerotic lesion formation with its high expression levels in atheroma plaques with the consequent accumulation of cholesterol [82] and during differentiation of human monocytes to macrophages with significant LD formation and TAG accumulation [83].

The role of Plin1 in foam cells is significant in the accumulation of lipids in the arterial wall, thus influencing atherosclerosis development [44]. Faber et al. found, for the first time, the upregulation of the Plin1 gene in ruptured human atherosclerotic plaques, observing a specific expression of both Plin1 mRNA and protein in the cytoplasm of several cells surrounding the cholesterol clefts, foam cells of the ruptured plaques, and endothelial cells of newly formed small vessels [84]. Their study paved the way for new potential regulators that may be involved in the destabilization of human atherosclerotic plaques, thus leading to a better understanding of the molecular processes involved in plaque destabilization.

Anyway, the role of Plin1 in macrophages is still unclear and controversial. Indeed, it was reported by Yamamoto et al. that the overexpression of Plin1 in macrophages also protects against atheroma progression in apolipoprotein (Apo) E knockout (KO) mice, probably reducing pro-inflammatory macrophages in unstable atheroma plaques [59]. The atherosclerosis decrease was also associated with a macrophage polarity change in plaque, following the high expression levels of Plin1. Therefore, their study demonstrated a direct effect of Plin1 on macrophages, and its higher concentration level had an atheroprotective role, maybe due to modification of the inflammatory profile and stability of macrophages. Additional investigation should be performed to clarify the mechanisms underlying the suppression of TG and CE storage in LDs, followed by a reduction in inflammation in plaques.

In addition, the role of LD proteins, including Plin1, in human macrophages was also recently investigated in human carotid atherosclerotic plaque samples and cultured macrophages derived from peripheral blood monocytes [81], demonstrating that Plin1 expression was associated with plaque stability and the anti-inflammatory phenotype, whereas Plin2 was primarily expressed in arterial plaques in symptomatic stroke patients and was related with the inflammatory potential of macrophages. Therefore, Plin1 showed atheroprotective effects, while Plin2 promoted the progression of atherosclerotic lesions. The study suggests that Plin1 might be responsible for a decrease in the inflammatory potential of macrophages that are components of more stable atheroma plaques.

In conclusion, several studies have shown the controversial role of Plin1 in atherosclerosis, which has been proposed as proatherosclerotic by increasing lipid storage in LDs and stimulating foam cell formation, and atheroprotective by lowering pro-inflammatory macrophages that produce unstable atheroma plaque.

Plin1 was originally described in adipocytes, but over the years, its involvement in atheroma plaque formation has also been described, suggesting its role in CVDs. Recently, Desgrouas et al. performed a systematic literature review aiming to better understand the pathogenicity of *PLIN1* variants in humans based on the variants’ locations within the protein and their types [44]. Anyway, further studies are needed to elucidate the physiopathological mechanisms of these variants and confirm Plin1 as biomarker for the prevention of CVDs.

### 3.3. Role of Plin1 in Cardiometabolic Disorders

As the comprehension of cardiovascular health deepens, the significance of the intricate inter-relationship between metabolic dysregulation and CVDs is becoming increasingly apparent [85]. Thus, the study of metabolic disorders could be fundamental in the understanding of molecular mechanisms at the basis of CVDs.

Plin1 deficiency mainly leads to reduced adiposity, aberrant adipocyte lipolysis, and metabolic disorders both in rodents and humans [48]. It has been observed that adipose tissue dysfunction may have a negative impact on the heart with a greater propensity to develop hypertrophic cardiomyopathy. It is well known that oxidative stress and lipotoxicity are the main causes of myocardial lesions and metabolic cardiomyopathies in obese and diabetic individuals [16]. The adipose tissue dysfunction in Plin1 null (Plin1^−/−^) mice resulted in excessive FA transport and β-oxidation, lipotoxicity, high levels of oxidative stress, mitochondrial abnormalities, and disorganized myofilament in cardiomyocytes [48]. Therefore, the cardiomyocytes in Plin1^−/−^ mice showed an alteration of the antioxidant capacity to remove excess ROS, thus leading to significant oxidative stress, which was also confirmed by reduced levels of the antioxidant glutathione (GSH) and the GSH/glutathione disulfide (GSH/GSSG) ratio. In addition, Plin1^−/−^ mice showed cardiac hypertrophy, leading to heart failure with left ventricular diastolic dysfunction at 20 weeks of age. The study highlighted a significant association between adipose tissue dysfunction and impaired lipid metabolism, lipotoxicity, and oxidative stress lesions in the Plin1^−/−^ mouse heart. All these changes were responsible for the development of hypertrophic cardiomyopathy and failure in these mice, which could explain the relevant complications occurring in two typical adipose tissue disorders in humans, i.e., lipodystrophy and obesity.

Omics techniques might play a crucial role in the study of WAT development and metabolic activity. In a 2021 study, Backdahl et al. [86] employed spatial transcriptomics to analyze a cellular WAT subpopulation ex vivo, directly studying the whole tissue without a homogenization process. Spatial transcriptomics is a technique that employs spatially barcoded poly-T capture probes to facilitate RNA sequencing from intact tissue sections, thereby preserving histological information [87]. This methodology has recently been utilized to uncover cellular heterogeneity and spatial organization within tumors and in the central nervous system [88,89,90,91]. They defined the microarchitecture of WAT by developing and applying algorithms that identified homo- and heterotypic cell clusters, which revealed that there were three different types of adipocytes with distinct transcriptional profiles and spatial arrangements, each characterized by a defined marker gene [86]. Indeed, *LEP* (encoding the adipokine leptin), *PLIN1/PLIN4* (encoding the LD-associated proteins Plin1 and 4), and *SAA1/SAA2* (encoding the serum amyloid A1 and A2 acute phase proteins) were the most distinctive genes for the respective classes. The group of adipocytes characterized by an enhanced expression of Plin proteins resulted in being the only one transcriptionally responsive to insulin stimulation, suggesting that the overall capacity of WAT to respond to insulin is determined by the proportion and function of a specific adipocyte subtype and, consequently, also to Plin expression.

### 3.4. Plin1 and Prenylcysteine Oxidase 1: A Novel Couple with a Strong Potential in Obesity-Related Disorders

The excess fat accumulation in WAT is associated with the development of metabolic syndromes such as CVDs and type 2 diabetes (T2DM) [92]. A better understanding of the molecular mechanisms underlying adipocyte differentiation is crucial for preventing obesity and metabolic diseases. Recently, it has been observed that the inactivation of prenylcysteine oxidase 1 (Pcyox1), which is a protein involved in the metabolism of prenylated proteins and actually known as a novel regulator of adipogenesis, affected the expression of several adipogenic mediators negatively, including Plin1 [93]. As already mentioned, Plin1 is mainly expressed in mature adipocytes and, in particular, on LD surfaces during the differentiation of 3T3-L1 adipocytes into lipid-accumulating mature adipocytes. Plin1 was less abundant in differentiated 3T3-L1 cells after Pcyox1 silencing [93]. Therefore, Pcyox1 regulates the key factors that control the adipogenic process, such as Plin1, and the targeted deletion of the *Pcyox1* gene in mice prevented lipid accumulation and adipose tissue inflammation. In addition, Plin1-KO mice exhibited high basal lipolysis because of the loss of the protective function of Plin on LDs, thus resulting in a reduction in TG deposits, glucose intolerance, and resistance to diet-induced obesity [94]. All these data confirmed the key role of Plin1 in LD formation and TG metabolism, together with Pcyox1, which could be an important regulator and potential therapeutic target for obesity-related disorders.

## 4. Perilipin 2

### 4.1. Structure and Functions of Plin2

Plin2 is a protein of 437 amino acids that includes several conserved regions, including a PAT domain (residues 9–100), an 11-mer repeating unit within residues 101–192, and two LD-targeting domains located in the middle and C-terminal regions [33,95]. In addition, the prediction of the Plin2 secondary structure highlighted that the C-terminal region contains an α-β domain, a four-helix bundle, and two β-strands, which form a cleft highly conserved in the Plin family and represent a lipid-binding site.

Perilipin 2 is the only constitutive and ubiquitously expressed LD protein [96] and the major human macrophage foam cell LD coat protein [62]. Plin2 is involved in both the biogenesis and catabolism of LDs. Plin2 overexpression protects LDs against autophagy-mediated degradation, whereas Plin2 under regulation promotes lipophagy and TAG catabolism [96]. In addition, the selective degradation of Plin2 from LDs by chaperone-mediated autophagy (CMA) allows the enrolment of cytosolic lipases and macroautophagy effectors to LDs, thus facilitating lipolysis [97]. The phosphorylation of Plin2 by the AMP Protein Kinase (AMPK) allows its dissociation from LDs after the interaction of Plin2 with the chaperone HSPA8/Hsc70 for LD catabolism. Unlike Plin1, Plin2 has minimal control over lipolysis and is not phosphorylated by PKA [35]. Indeed, Plin2 does not regulate the access of lipases and their co-factors to LDs through protein binding interactions, whereas the upregulation of Plin2 slows the recruitment of ATGL, ABHD5, or HSL to LDs under either basal or lipolytically stimulated conditions, thus reducing lipolysis [96].

In recent years, numerous in vitro and in vivo studies focused attention on Plin2 due to a close association between changes in its expression and alterations of intracellular lipid metabolism, impaired FA uptake, and LD formation in different types of cells, including mice and humans [98]. Plin2 is not only an intracellular protein but it is also expressed in body fluids as a circulating protein both in urine and plasma; especially higher amounts have been observed in cancer patients in comparison to healthy subjects [99,100,101]. Moreover, Plin2’s association with health parameters and body composition was recently evaluated by Conte et al., demonstrating that circulating levels of Plin2 were significantly higher in women than men, in particular, in advanced age (60–79 years) but not in old age and centenarians, and positively correlated with fat mass, inflammatory, and metabolic parameters [98]. Previously, it was also reported that four Plin proteins in human skeletal muscle, including Plin2, had more increased concentrations in women than men, confirmed by a higher intramyocellular lipid content observed in female skeletal muscle [102].

In addition, a recent paper by Scorletti et al. [103] investigated the effect of a missense variant in human Plin2 (*PLIN2 Ser251Pro*) on the reduction in hepatic steatosis. In human patient *PLIN2-Pro251* carriers, they demonstrated that this specific polymorphism was associated with a decreasing trend in liver fat, as diagnosed by magnetic resonance spectroscopy, in males but not in females. There was also a significant association between *PLIN2-Pro251* and a reduction in visceral adipose tissue in all individuals; however, this was not confirmed when considering males and females separately. Anyway, the sex stratification of the lipidomic profile of *PLIN2-Pro251* carriers highlighted a difference in males compared with females, proving higher lipid secretion and lower lipid accumulation in the liver [103].

Therefore, changes in the characteristics of Plin proteins have been evaluated over the years with interest in connections to gender, age, obesity, and other perturbations.

In 2003, Wang et al. [104] discovered greater Plin1 protein concentration, which is the major Plin expressed in adipose tissues, in subcutaneous adipocytes from males with obesity compared to women. In addition, later, Ray et al. stressed the importance of studying depot-specific differences in Plin expression either in men or in women, thus evaluating gender differences [105].

### 4.2. Role of Plin2 in Atherosclerosis and CVDs

It is widely recognized that the overexpression of Plin2 plays a significant role in LD accumulation across various tissues, including the heart [40]. Plin2 is the most upregulated Plin protein present in atheromas. Indeed, Plin2 plays a significant role in the lipid metabolism of macrophages in atherosclerotic plaques and enhances plaque inflammation [47]. In ApoE-deficient mice (ApoE^−/−^), Plin2 promoted foam cell formation and its inactivation led to less formation of LDs and protection from atherosclerosis [60].

In addition, high levels of Plin2 in human atherosclerotic lesions are associated with plaque instability, as demonstrated by immunohistochemical analyses of samples of unstable plaque, where a higher Plin2 expression was observed with a correlated increased protein kinase C (PKC) expression [106]. According to these data, Plin2 mRNA was also significantly enhanced in unstable plaque tissue compared to its expression in stable plaque and in normal control tissue.

Plin2 is also able to increase pro-inflammatory cytokine expression and secretion, such as tumor necrosis factor-alpha (TNF-α), monocyte chemoattractant protein-1 (MCP-1), and interleukin (IL)-6, in THP-1 macrophages [107]. Indeed, the inhibition of Plin2 expression in THP-1 monocytes is protective against atherogenesis by reducing pro-inflammatory gene expression through TG-rich lipoprotein lipolysis products and increasing the size of LDs [61].

Several studies have shown the involvement of lipophagy in regulating cellular lipid levels in various tissues and disease conditions [108,109]. It is well known that Plins regulate lipophagy and cholesterol efflux in macrophage foam cells. Indeed, selective macrophage autophagy is a powerful anti-atherogenic activity that promotes the degradation of cytosolic LDs to preserve cellular lipid homeostasis [62]. Since impaired lipophagy may be the primary mechanism driving several diseases, such as obesity, fatty liver, atherosclerosis, and metabolic syndrome [110], it is important to study lipophagy factors and their relationship with LD decomposition to elucidate the pathogenesis and treatment of cardiometabolic disorders. Mass spectrometry (MS) was successfully employed to characterize the human macrophage foam cell LD proteome and identify lipophagy selectivity factors [62]. Differentiated human THP-1 macrophages were incubated with aggregated low-density lipoprotein (agLDL) to promote LD and foam cell formation and cultured under basal conditions or upon autophagy inhibition by chloroquine [62]. Several structural LD proteins, such as Plin2, Plin3, and vimentin, were identified together with other LD-specific proteins involved in lipid transport and metabolism, lipid hydrolysis, vesicular transport, lysosomal function, ubiquitination, and autophagy regulation. Moreover, transcriptome profiling has shown that *PLIN2* was significantly increased in macrophage foam cells, which are a hallmark of atherosclerosis, in comparison to macrophages without foam cells. Since the expression of these identified lipophagy factors is altered in macrophage foam cells, contributing to the process of atherosclerosis development, the possibility to selectively target them could be a potential therapeutic strategy to treat atherosclerosis through the increase in the lipophagy and the stimulation of reverse cholesterol transport and LD catabolism [62]. Therefore, promoting lipophagy could be useful in the treatment of heart diseases and other metabolic disorders. In particular, myocardial ischemia is closely linked to impaired metabolism and lipid overload [111]. Plin2 is expressed on myocardial LDs, and it has been observed that its higher cardiac levels resulted in massive myocardial steatosis [112]. Anyway, contrary to the expectations, an increased lipid accumulation in cardiomyocytes was also reported in Plin2^−/−^ mice, even if they had normal cardiac function both under baseline conditions and after dobutamine-induced stress [57]. In addition, these higher levels of TGs were not reported in cardiac fibroblasts, suggesting a cardiomyocyte-specific increase in lipid storage [57]. Following an induced myocardial infarction, cardiac function was reduced in comparison to Plin2^+/+^ mice. The increased TG accumulation in the heart of Plin2-deficient mice was not due to abnormal mitochondrial respiration, which was preserved, but it was caused by reduced lipophagy, suggesting that Plin2 plays an important role in the regulation of LD hydrolysis [57]. Further studies are essential to clarify the link between Plin2 deficiency and lipophagy or lysosomal activities, but Plin2 involvement in lipophagy induction is evident.

An increased abundance of Plin3 and Plin5 was also observed in Plin2^−/−^ hearts, which were primarily co-localized with LDs throughout the cardiomyocyte [57]. It is not yet clear whether this is a compensatory event or a consequence of lipid accumulation. However, these data confirm a close interplay between Plins, whose concentrations regulate lipase access to the lipid pool and thus LD hydrolysis. Therefore, not only the number of LDs but also their protein composition is crucial in the myocardial function.

Recently, the involvement of Plin2 has also been studied in coronary microvascular obstruction (MVO) in STEMI patients undergoing primary percutaneous coronary intervention, showing that Plin2 levels were increased in patients with MVO and independently associated with MVO and infarct size [47]. Plin2 was also an independent predictor of major adverse cardiovascular events at a mean clinical follow-up of 6 months, suggesting a possible role of Plin2 as a target of future therapies. The higher expression of Plin2 causes an increase in LD accumulation in foam cells at atherosclerotic plaques, thus reducing their degradation and cholesterol efflux processes [113]. The overload of extracellular free cholesterol due to cell death can lead to cholesterol crystallization in the necrotic core of atherosclerotic advanced lesions with intimal injury and destabilization of atherosclerotic plaque [114]. Therefore, Plin2 could be responsible for plaque rupture, resulting in STEMI or other cardiovascular pathologies [47]. The overexpression of Plin2 may cause a rapid progression of coronary atherosclerosis, accelerating plaque growth, local vulnerability, and inflammation, as also confirmed by other studies [113,115,116], and stimulate increased production of ROS and greater release of metalloproteinases after plaque rupture.

A study by Sato et al. [117] revealed that the cardiac-specific overexpression of Plin2 in aged mice induced atrial steatosis, characterized by the accumulation of LDs in atrial cardiomyocytes and elevated atrial TAG levels. This steatosis led to electrical remodeling, including the lateral redistribution of connexin 43 (Cx43) and impaired intercellular communication, resulting in slower and heterogeneous conduction propagation. These changes predisposed to atrial fibrillation (AF) in aged Plin2-transgenic mice [117]. Furthermore, Plin2-induced atrial steatosis exacerbated the effects of obesity and metabolic disorders on cardiac remodeling. The exclusive accumulation of specific lipid species, such as TAG and DAG, suggested a lipotoxic mechanism contributing to Cx43 remodeling and AF susceptibility. Importantly, TAG depletion in double-transgenic mice overexpressing both Plin2 and HSL reduced lipid accumulation and ameliorated AF susceptibility, implicating that steatosis is a key driver of the arrhythmogenic phenotype [117]. Their study underscores the role of Plin2 and cardiac lipid dysregulation in structural and electrical remodeling, providing insights into how obesity and metabolic disorders enhance AF risk.

### 4.3. Role of Plin2 in Cardiometabolic Disorders

Plin2 is involved in LD formation in the liver and peripheral tissues. A study on the function and expression control of Plin2 during adipogenesis of mouse embryonic fibroblasts, which have good physiological characteristics closer to in vivo adipocytes, revealed that protein levels of Plin2 did not change during adipocyte differentiation, although Plin2 mRNA levels increased [118]. In addition, the expression of Plin2 is lower in fully differentiated adipocytes than that of Plin1, which shows a higher binding affinity for LDs, thus becoming the major LD-associated Plin in mature adipocytes at the expense of Plin2, which is more prone to degradation [35]. Therefore, when adipocytes are lipolytically stimulated, there is a massive TAG hydrolysis with the consequent formation of new LDs. Plin2 has a positive key role in adipocytes during lipolysis, binding the LD fraction and becoming stable, thus escaping proteasomal degradation [118]. Instead, its cytosolic form is more susceptible to ubiquitination and degradation mediated by the N-terminal region.

Moreover, recent studies revealed that the p38/MAPK pathway is involved in the regulation of poly-unsaturated FAs, TAGs, and LD synthesis, also modulating the cellular levels of Plin2. Indeed, Li et al. [119] demonstrated that Plin2 is highly expressed in apoptotic cells and that the inhibition of p38/MAPK affects the mRNA levels of Plin2 and other Plins, consequently affecting LD formation. The levels of Plin2 modulate correspondingly to TAG and LD depletion.

The accumulation of TAGs in the liver may be associated with the progression of non-alcoholic fatty liver disease (NAFLD), a condition characterized by increased levels of TAGs in more than 5% of hepatocytes in the absence of excessive alcohol consumption [120]. Plin2 is the most abundant protein in hepatic LDs.

The identification of Plin2 as a potential target in metabolic liver disorders was reported by different studies. Moreto et al. [121], studying the livers of rats affected by NAFLD, demonstrated by proteomic analysis that Plin2, together with ApoE, is highly downregulated in animals treated with carnosine, a natural dipeptide with several protection roles against oxidative stress. Wang et al. [122] used quantitative proteomics analysis based on tandem mass tag labeling coupled with liquid chromatography–tandem mass spectrometry (LC-MS/MS) to study the livers of NAFLD-affected mice treated with a high-fat diet (HFD). Their findings revealed that the administration of silibinin, a natural herb that originated in Southern Europe and North Africa utilized to treat liver diseases, can decrease the levels of Plin2 in the liver of treated animals, decreasing liver lipid deposition and increasing insulin sensitivity.

To study metabolic-associated fatty liver disease (MAFLD), an inflammatory condition that can progress to cirrhosis and hepatocellular carcinoma and the leading indication for liver transplantation, Ichikawa et al. developed a fatty liver mouse model that more closely approximates the pathophysiology of MAFLD in humans [123]. The animal model generated was unique since various degrees of fatty liver could be induced depending on the type of diet and the use of growth hormone supplementation. Liver tissues were analyzed by different omics methods to elucidate the pathological conditions and examine the drug efficacy, including transcriptomics, proteomics, and metabolomics. Moreover, they performed a multi-omics analysis providing a method for the joint multi-omics analysis of up to three datasets. Although the independent analysis of individual omics datasets is crucial, joint analysis offers unique insights and reveals trends that resonate across two or more omics datasets, despite the statistical and combinatorial challenges involved. Utilizing this integrated approach, MiBiOmics identified Plin2 as a co-inertia driver, indicating its potential as a target for the development of treatments for MAFLD. Plin2 has been identified as a key player in the initiation and progression of MAFLD [42]. Notably, the deletion of Plin2 in a mouse model resulted in decreased TAG and cholesterol levels in the liver, which was attributed to the suppression of the genes involved in lipogenesis and cholesterol biosynthesis [124].

In conclusion, Plin2 is an LD protein with various metabolic functions, and it may be a useful marker of metabolic and cardiovascular disorders, thus predicting critical illness outcomes.

## 5. Perilipin 3

### 5.1. Structure and Functions of Plin3

The Plin3 protein has a sequence of 434 amino acids and shares with the other Plin proteins a conserved protein domain architecture: an N-terminal PAT domain, an 11-mer repeat region, and a C-terminal four-helix bundle [125]. Recently, it has been shown that the N-terminal PAT domain of Plin3 binds DAG, the immediate precursor to TAG, thus promoting LD formation at the membrane of the ER [126]. Indeed, Khaddaj et al. also confirmed that Plin3 promotes the early stages of LD biogenesis, stimulating the development of ER membrane domains enriched with DAG [127]. Therefore, Plin3 seems to have membrane-organizing properties by localizing DAG within the ER membrane bilayer and enhancing LD synthesis. Plin3 and DAG cooperate within the ER membrane and induce the localization of LD biogenesis proteins into ER subdomains.

Plin3 is ubiquitously expressed [37] and has similar functions to Plin2, which limits lipase access to the LD core. In addition, Plin3 is a CMA substrate like Plin2, which mediates its degradation strictly regulated by AMPK [128].

Plin3 has been minimally studied for its role in controlling lipolysis [35]. However, Plin3 seems to play an important function in obesity, promoting the transition from brown to white adipose tissue [37], and in cancer pathogenesis [54,129,130].

### 5.2. Involvement of Plin3 in Atherosclerosis and CVDs

Plin3 is relatively understudied compared to other Plin proteins in the cardiovascular field. Anyway, Plin3 is primarily involved in TAG accumulation in macrophages and preserves TAG levels when macrophages are depleted of Plin2 [65]. Moreover, Plin3 expression is directly enhanced by the combination of elevated serum levels of glucose, insulin, and free FAs, which are major risk factors promoting atherosclerosis or CVDs.

These data confirmed a significant Plin3 involvement in the conversion of macrophages into foam cells. Plin3 appears to stimulate LD expansion during foam cell formation rather than their stabilization [116]. Therefore, Plin3 could be another potential molecular target for the prevention or therapy of atherosclerosis.

In this regard, the literature data are conflicting. While some authors have observed that Plin3 is highly expressed in macrophage-derived foam cells and implicated in foam cell formation [65,66], others have described a reduction in Plin3 expression in a foam cell model based on the oxidized low-density lipoprotein (ox-LDL) in macrophages originating from the THP-1 cell line using microarrays [63].

In vitro studies showed a role of Plin3 in the development of atherosclerotic lesions, although more detailed research is needed to clarify the location and expression level of this protein in atherosclerotic lesions. Higher Plin3 levels were found in advanced atherosclerotic plaques associated with human lipid-rich vascular smooth muscle cells (VSMCs), and Plin3 was mainly located in cellularized regions around the necrotic core [64]. Therefore, Plin3 downregulation in VSMC might prevent their transition toward a foam cell phenotype and the formation of vulnerable atherosclerotic plaques.

### 5.3. Role of Plin3 in Cardiometabolic Disorders

LDs in the liver are covered with Plin proteins, in particular, Plin2 and Plin3, but the available literature concerning the characterization of Plin3 in the cardiometabolic context is limited.

It has been assumed that the reduction in Plin3 via antisense oligonucleotide treatment in mice attenuates hepatic steatosis, reducing hepatic and serum TAG levels and improving glucose tolerance and insulin sensitivity [131]. Moreover, the inhibition of Plin3 expression in HeLa cells affected LD maturation with a decreased incorporation of TAG into LDs [6]. Instead, the suppression of Plin3 in mouse AML12 hepatoma cells did not reduce TAG accumulation, but the LDs were smaller and mainly covered with Plin2. Indeed, AML12 cell LDs contained Plin2 at the surface, while Plin3 was found in both cytosolic and LD compartments [132]. Therefore, the downregulation of Plin3, Plin2, or both induced changes in LD morphology and surface profiles. The absence of both PAT proteins led to a drastic increase in LD size and decrease in LD number, maybe due to the fusion of LDs that minimizes surface area in contact with the surrounding cytosol [35].

In humans, a study was performed to determine Plin3 levels among adults with varying degrees of obesity and insulin resistance, aiming to define the cardiometabolic associations of this protein [133]. Circulating Plin3 was significantly lower in obese patients with T2DM in comparison to healthy and lean subjects. In addition, the levels of TGs, total cholesterol, glucose, and insulin were inversely correlated with Plin3 in all subjects. Therefore, circulating Plin3 could be a potential predictive biomarker for T2DM as it is significantly associated with insulin resistance indices.

## 6. Perilipin 4

### 6.1. Structure and Functions of Plin4

Plin4, also known as the adipocyte protein S3-12, is primarily expressed in WAT, with lower concentrations found in skeletal muscle and the heart, while it is undetectable in the liver.

The *PLIN4* locus gene is characterized by significant polymorphisms. Two specific intron polymorphisms, rs8887 and rs884264, are associated with increased adiposity [134]. Additionally, the latter variant has been proposed as a miRNA regulatory site, potentially leading to a reduction in Plin4 expression [135].

The Plin4 protein contains 1371 amino acids and a specific region of 27 × 33 amino acid approximate tandem repeats. The protein structure of Plin4 is distinct from that of other Plin isoforms [136]. It lacks the PAT domain in the N-terminal region of about 100 amino acid residues and features an unusually long and repetitive amphipathic helix. Specifically, this repeated region contains approximately 90 repeats, whereas the other Plin isoforms have only 7–9 repeats, which are less conserved and often interrupted by insertion or deletions [137].

Plin4 plays a crucial role in the packaging of TAG into adipocytes and is involved in the biogenesis of LDs as a coat protein. Additionally, it is present freely in the cytoplasm of LDs. Plin4 associates with the surface membrane of nascent LDs, promoting their stability. Its expression is stimulated by the activation of peroxisome proliferator-activated receptor gamma (PPARγ). In WAT, Plin1 and Plin4 are transcriptionally activated through the binding of PPARγ to their respective promoter elements [138].

This isoform of the Plin family is predominantly expressed in pre-adipocytes, like Plin3, with its levels substantially increasing after adipocyte differentiation. Although Plin4 is relatively specific to adipose tissue, its deletion in Plin4^−/−^ animals does not affect the development or maintenance of adipose tissue. Moreover, the Plin4 gene is located directly downstream of the Plin5 gene, and the silencing of *PLIN4* was linked to a concurrent decrease in the expression of Plin5, as demonstrated by Chen et al. in their research involving a Plin4^−/−^ mouse model [139]. Furthermore, the deletion of *PLIN4* resulted in a reduction in cardiac TG accumulation under basal conditions and after dietary treatment.

Plin4 is probably the less investigated member of the Plin family in the cardiovascular field. This protein is not expressed in mouse macrophages or atherosclerotic lesions, differently from the other isoforms Plin1, Plin2, and Plin3, and no studies were conducted in human samples [116,140].

### 6.2. Role of Plin4 in Cardiometabolic Disorders

As mentioned above, the Plin4 isoform is highly abundant in WAT and plays a key role in LD formation and lipid metabolism. The Plin4 protein is also involved in fat distribution and metabolic dysfunction. Koprulu et al., combining the power of the genome-wide analysis of array-based rare, nonsynonymous variants in 450,562 individuals in the UK Biobank with exome sequence-based rare loss of function (LoF) gene burden testing in 184,246 individuals, revealed that the LoF of *PLIN4* had metabolic adverse effects. Indeed, *PLIN4* LoF affected the body mass index, TGs, and HDL-cholesterol levels, suggesting the implication of this gene in the regulation of fat distribution and identifying it as a potential target for this disorder [141].

## 7. Perilipin 5

### 7.1. Structure and Functions of Plin5

Plin5, the most recent member of the Plin family, is predominantly expressed in highly oxidative tissues and plays a pivotal role in coordinating FA and glucose oxidation within cardiomyocytes.

Plin5 is a protein of 463 amino acids with a structure that is homologous at the N-terminal region to that of the other members of the Plin family but contains, at the C-terminus, a unique domain that confers to Plin5 the ability to recruit LDs to mitochondria [142]. Indeed, Plin5 is recognized as a key candidate for mediating the physical interactions between LDs and mitochondria in oxidative tissues [143]. The overexpression of Plin5 enhances the close association between LDs and mitochondria [144]. These interactions, facilitated by Plin5, are thought to promote the efficient channeling of FAs from LDs to mitochondria in a manner that is both energetically and redox-controlled. This process is likely regulated by PKA signaling, along with the subsequent modulation of interactions between ATGL and comparative gene identification-58 (CGI-58) [145].

Plin5 directly interacts with ATGL [146], but, similar to Plin1, it can also interface with CGI-58, which acts as a coactivator for both ATGL and HSL [74]. While Plin5 has only a minimal effect on ATGL activity in vitro when free triolein is used as a substrate [147], ATGL-mediated lipolysis is notably diminished when utilizing Plin5-coated LDs, compared to those coated with Plin2-LDs [148]. Consequently, it is hypothesized that Plin5 acts as a regulated lipolytic barrier, limiting access to LD substrates for ATGL [144]. Experiments examining different levels of Plin5 in relation to ATGL and CGI-58 suggest that Plin5 restricts lipolysis by disrupting the interaction between ATGL and its activator, CGI-58. This interaction appears to recognize a binding motif on Plin5 that is distinct from that on Plin1 [146]. In vitro studies show that Plin5-coated LDs provide stronger protection against hydrolysis mediated by CGI-58 and demonstrate a greater increase in activity upon PKA activation compared to Plin1-coated LDs [148].

It has been demonstrated, both in vivo and in vitro, that Plin5 is also a substrate for PKA phosphorylation [149]. Plin5 contains a single PKA phosphorylation site at serine 155. Mutations at this site (serine 155A) suggest that PKA plays a crucial role in regulating LD hydrolysis in oxidative tissues, similar to the function of Plin1 [148].

The precise molecular mechanisms by which Plin5 regulates LD storage are still not fully clarified. Nonetheless, it is proposed that the phosphorylation of Plin5 at serine 155 by PKA may trigger a structural reconfiguration of Plin5 on the surface of LDs. This reorganization likely facilitates the assembly of ATGL and CGI-58, thereby increasing the accessibility of HSL to lipid substrates, independent of Plin5 expression levels [150]. Interestingly, interactions between ATGL and CGI-58 are enhanced in contracted skeletal or cardiac muscle cells compared to resting cells, without disrupting the association of Plin5 with either ATGL or CGI-58 [151].

Unlike Plin1 and 2, Plin3, Plin4, and Plin5 demonstrate greater stability even in the absence of binding to cytosolic LDs and can interact with non-LD components within the cytosol [152]. Imaging and cellular fractionation studies have revealed that Plin3, Plin4, and Plin5 are organized into high-density, nonmembrane disk structures. When there is an increase in the synthesis of TAGs or CEs, Plin3, Plin4, and Plin5 quickly re-localize to sites of LD formation [6,153].

Plin5 is prominently expressed in BAT, whereas its levels are barely detectable in WAT.

Plin5 expression is upregulated under both physiological and pharmacological conditions that lead to systemic increases in FAs, with effects that are specific to different cell types. For instance, this upregulation occurs in the liver and heart during fasting, in skeletal muscle during endurance exercise, and in the liver following chronic β3-adrenergic stimulation [31,154]. Basal levels of Plin5 mRNA in tissues such as the liver and heart are significantly reduced in PPARα^−/−^ mice. However, fasting induces Plin5 expression in these mice, indicating the presence of additional regulatory mechanisms. Notably, PPARβ/δ has a more pronounced regulatory effect on Plin5 expression in skeletal muscle compared to PPARα [155]. Plin5 expression can also be induced in cell cultures by exogenous FAs and in the liver, as well as in skeletal and cardiac muscles, through PPARα agonists. Additionally, the PPARγ agonist pioglitazone can induce Plin5 expression in WAT [155,156].

### 7.2. Role of Plin5 in Atherosclerosis and CVDs

The tissue distribution of Plin5 highlights its essential role in regulating LD functions within the heart and blood vessels. This regulation is critical for optimal myocardial cell function, the formation of foam cells, and the progression of atherosclerosis [15].

Plin5 is an important regulator of oxidative stress, which is significantly involved in atherosclerosis development, and its knockdown in animal models enhanced inflammation, apoptosis, oxidative stress, and lipid accumulation, thus promoting atherosclerosis progression [45]. In addition, Plin5 was also demonstrated to be involved in vascular disorders, such as microvascular endothelial dysfunction and atherosclerosis [69]. MS was recently applied to investigate the interactome of Plin5, whose cardiac-specific overexpression is involved in cardiac physiological hypertrophy stimulation [157]. Since it is still unclear whether physiological hypertrophy has a cardioprotective role in improving cardiac metabolism and oxidative functions, a greater understanding of the underlying pathways could be useful for the identification of novel therapeutic targets for CVDs. Indeed, cardiac hypertrophy is an adaptive response to cardiac stress, but the pathological progress of hypertrophy often leads to heart failure [158]. High levels of Plin5 were observed in several previous studies as a protective factor for heart function after cardiac stress [58,68]. To clarify how elevated cardiac Plin5 promotes physiological cardiac hypertrophy, co-immunoprecipitation and LC-MS were performed on protein lysates from primary MHC-*Plin5* (transgenic mice with cardiac-specific expression of FLAG-tagged *Plin5)* and wild-type cardiomyocytes [157]. More than 400 proteins were identified in the FLAG-enriched fraction, among which 79 were overexpressed in lysates from MHC-*Plin5* in comparison to wild-type cardiomyocytes. In particular, sarcoplasmic/endoplasmic reticulum Ca^2+^ ATPase 2 (SERCA2) was one of the strongest Plin5-interacting partners, and the formation of Plin5/SERCA2 protein complexes was observed in cardiomyocytes to stimulate calcium handling and cardiac muscle contraction. Therefore, high Plin5 has a beneficial role in the regulation of cardiac contractility and calcium handling through the modulation of SERCA2 function [157].

Previous studies have also shown the important role of Plin5 in myocardial function and lipid metabolism [58]. Both *Plin5*-null and overexpression models clearly showed that Plin5 is essential for normal cardiac functions and metabolism [159], but above all, the higher expression of Plin5 is central to cardiomyopathy development [144,160]. Plin5 is crucial for preventing excessive lipolysis and keeping LDs at detectable sizes in the heart so that LDs limit excess ROS generation by protecting FAs from oxidation, thus reducing the impact of oxidative stress on the heart [67]. Indeed, in *Plin5*-null mice hearts, the production of ROS was enhanced, causing rapid deterioration of heart function with age. The administration of N-acetylcysteine alleviated oxidative stress, preventing the heart’s functional decline [67].

Using lipidomics to analyze the heart tissues, a dramatic decrease in TG and DAG levels was observed in Plin5^−/−^ mice compared to wild-type mice, and the heart function was reduced following myocardial ischemia or stress [58]. This opposite effect of Plin5 deficiency on lipid content in the heart in comparison to Plin2 is related to the important role of Plin5 in the association between LDs and mitochondria, which was markedly lower in Plin5^−/−^ mice. Indeed, LDs were smaller and fewer than those present in wild-type mice and not closely located to mitochondria in the heart of Plin5^−/−^ mice. Low TG storage due to Plin5 deficiency resulted in reduced FA uptake and increased glucose uptake to preserve energy metabolism under physiological conditions. After myocardial ischemia, FA oxidation was downregulated in Plin5^−/−^ hearts, and changes were evident in the left ventricular diastolic volume, stroke volume, and ejection fraction [58]. Therefore, these results demonstrated that Plin5 has a cardioprotective role following ischemia. This role was also confirmed by the analysis of a human cohort with clinically suspected coronary artery disease that showed a common genetic variation of *PLIN5*. This SNP led to reduced *PLIN5* cardiac expression, impaired heart function, and adverse outcomes after myocardial ischemia, but it did not affect *PLIN4* expression [58].

### 7.3. Role of Plin5 in Cardiometabolic Disorders

Plin5 offers a promising therapeutic target for metabolic disorders affecting the heart. By coating LDs in cardiomyocytes, Plin5 is closely associated with the production of ROS originating from cardiac mitochondria, which, in turn, influences the progression of diabetic cardiomyopathy [161]. Notably, diabetic Plin5^−/−^ mice showed reduced myocardial lipid accumulation, ROS production by nicotinamide adenine dinucleotide phosphate oxidase, and DAG/ceramide-PKC pathway, thus preventing type 1 diabetes-induced heart malfunction [162].

Heart function and energy metabolism were also studied in Plin5-Tg mice fed a HFD, which showed a cardiomyocyte-specific Plin5 overexpression with abnormal cardiac TAG accumulation, reduced lipolysis, and FA oxidation [163]. Cardiac TAG overload and increased LV mass are a typical feature of obesity and T2DM and highly correlate with heart morbidity and mortality [163]. These mice showed cardiac hypertrophy and adverse cardiac remodeling on HFD with heart function that declined with age due to prolonged exposure to HFD-induced metabolic stress. They also had reduced body weight and adipose tissue mass, as well as protection from diet-induced glucose intolerance and insulin resistance. In addition, impaired cardiac energy catabolism increased adipose tissue ß-adrenergic signaling, adipocyte lipolysis, and thermogenic activity, which were able to prevent HFD-induced obesity. All these data remark on the importance of carefully controlled nutritional management, which is crucial for patients with heart steatosis and hypertrophy. Reducing cardiac lipolysis or FA oxidation can be therapeutic for cardiomyocytes under cardiac damage or reperfusion stress, thus promoting cardiac efficiency [163].

The involvement of Plin proteins in lipid metabolism and storage has been studied over the years in several models of hypertrophic cardiomyopathies and cardiac remodeling induced by conditions, such as obesity and pregnancy, or environmental factors.

Recently, for the first time, it was shown that during established pregnancy and the postpartum period in the rat heart, Plin1, Plin2, and Plin5 isoforms are differently upregulated along with peroxisome proliferator-activated receptor-gamma coactivator 1-alpha, a regulator of mitochondrial biogenesis and respiration [71]. These results suggested that, together, they may contribute to the regulation of cardiac lipid metabolism induced by pregnancy. As a result, cardiac total TAG and cholesterol content increased during pregnancy-induced physiological cardiac hypertrophy following the return to normal levels after this adaptation of the heart.

In addition, understanding the impact of environmental factors on CVD development and outcomes is also critical; thus, the goal of a recent study was to determine the effect of bisphenol S (BPS) exposure on HFD-induced heart remodeling [164]. It was observed for the first time that BPS promoted cardiac remodeling and aggravated HFD-induced cardiac hypertrophy, fibrosis, and inflammation in mice. Only HFD mice and HFD mice exposed to BPS showed cardiac steatosis with increased cardiac Plin5 protein expression in relation to mice on a standard chow diet. Therefore, the study of the impact of this compound on cardiac remodeling highlighted that the association with an HFD worsens the hypertrophic profile, and it will also be interesting to assess its effects on the heart taking sex differences into account.

## 8. Conclusions and Future Perspectives

Lipid metabolism disorder with abnormal intracellular lipid buildup can lead to metabolic diseases, such as diabetes, obese cardiomyopathy, or liver diseases, causing irreparable damage to the cardiovascular system. The LD proteome has been extensively studied over the years through several proteomics-based approaches and the Plin multiprotein family has been identified as the most abundant signature of LDs. Plin proteins have distinct tissue distributions, subcellular locations, and lipid-binding characteristics, indicating diverse cellular activities [159].

These proteins are prevalent on LD surfaces in both adipose and non-adipose cells, and they are involved in lipid storage and hydrolysis, as well as LD formation and expansion. Therefore, Plin proteins play an important regulatory role in LD tissue-specific lipid accumulation and arrangement.

Understanding the mechanisms regulating the formation and dynamics of LDs and how a large number of proteins affect their structure and activities have been important goals for many years, leading to several significant and exciting discoveries that improved our comprehension of metabolic disease pathogenesis. Considerable progress has been made in understanding, for example, the functions of cardiac LDs and the mechanisms that regulate them. The distribution tissue of Plin proteins across various cardiac tissues highlights their essential role in the regulation of LD functions within the heart. It is well known that in murine models, the modification of Plin2 expression significantly influences the amount of cholesteryl ester in macrophages and the level of atherosclerosis. In addition, the myocardium in mice is the tissue that expresses the highest levels of Plin5, also known as myocardial LD protein. The future challenge will be to translate the findings on Plin proteins from cell and animal models to human CVDs, evaluating the differences in susceptibility to atherosclerosis or cardiomyopathies based on gene variants in the Plin protein family.

The research on Plin proteins’ response to several pathophysiological conditions in vivo is only beginning, and further studies are necessary to provide a complete picture of their roles. To date, the study of Plin has highlighted a complex network of signaling and regulatory factors of Plin proteins and has provided insight into the physiological roles of LDs and their association with cardiometabolic diseases. However, intensive work will still be needed to delineate the specific roles of this protein family so that they can represent novel and important drug targets for therapy and prevention of cardiometabolic diseases.

## Figures and Tables

**Table 1:** Characteristics of perilipin isoforms. CVDs, cardiovascular diseases; DAG; diacylglycerol; ER, endoplasmic reticulum; FA, fatty acid; LD, lipid droplet; MAFLD, metabolic-associated fatty liver disease; PKA, protein kinase A; Plin, perilipin; ROS, reactive oxygen species; T2DM, type 2 diabetes mellitus; TAG, triacylglycerol.

Perilipin	Alternative Name	Genomic Location	Univocal Structural Characteristics	Role in CVDs	Role in Cardiometabolic Disorders
Plin1	Perilipin	15q26.1	- A total of 522 amino acids. - Five consensus sites for the phosphorylation of serine residues by PKA.	- High expression in atherosclerotic lesions leading to increased cholesterol levels. - Overexpressed in macrophages becoming foam cells, leading to TAG accumulation.	- Plin1 deficiency reduces adiposity and metabolic disorders in human and rodents.
Plin2	Adipophilin	9p22.1	- A total of 437 amino acids. - C-terminal region contains an α-β domain, four-helix bundle, and two β-strands, which represent a lipid-binding site.	- Plin2 is the most upregulated Plin in atheromas. - Plin2 plays a significant role in the lipid metabolism of macrophages in atherosclerotic plaques. - It enhances plaque inflammation. - High levels of Plin2 in human atherosclerotic lesions are associated with plaque instability. - Plin2 increases pro-inflammatory cytokine expression and secretion.	- During lipolysis, Plin2 in adipocytes binds LDs, becoming stable and escaping proteasomal degradation; its cytosolic form is more susceptible to ubiquitination and degradation. - The levels of Plin2 modulate TAG and LD depletion. - Plin2 has been identified as a key player in the initiation and progression of MAFLD.
Plin3	Tip47	19p13.3	- A total of 434 amino acids. - N-terminal PAT domain of Plin3 binds DAG, thus promoting LD formation at the membrane of the ER.	- Plin3 expression is directly enhanced by the combination of elevated serum levels of glucose, insulin, and free FAs, which are major risk factors promoting atherosclerosis or CVDs. - Plin3 is involved in the conversion of macrophages into foam cells. Plin3 appears to stimulate LD expansion during foam cell formation.	- Circulating Plin3 could be a potential predictive biomarker for T2DM, being significantly associated with insulin resistance indices.
Plin4	S3-12	19p13.3	- A total of 1371 amino acids. - Specific region of 27 × 33 amino acids approximates tandem repeats. - Lacks the PAT domain in the N-terminal region of about 100 amino acid residues. - Long and repetitive amphipathic helix, repeated region with approximately 90 repeats.	- Plin4 is the less investigated member of the Plin family in the cardiovascular field.	- Polymorphisms rs8887 and rs884264 are associated with increased adiposity. - Involved in fat distribution and metabolic dysfunction.
Plin5	Oxpatperilipin	19p13.3	- A total of 463 amino acids. - Contains, at the C-terminus, a unique domain that confers to Plin5 the ability to recruit LDs to mitochondria.	- High levels of Plin5 were observed in several previous studies as a protective factor for heart function after cardiac stress. - Plin5 is crucial for preventing excessive lipolysis and keeping LDs at detectable sizes in the heart so that LDs limit excess ROS generation by protecting FAs from oxidation, thus reducing the impact of oxidative stress on the heart. - Cardioprotective role following ischemia.	- Plin5 is closely associated with the production of ROS originating from cardiac mitochondria, which, in turn, influences the progression of diabetic cardiomyopathy.

**Table 2:** Comparative table of the Plins in different atherosclerosis-related conditions. agLDL, aggregated low-density lipoprotein; ApoE, apolipoprotein E; FA, fatty acid; HFD, high-fat diet; LD, lipid droplet; MHC, α-myosin heavy chain; ROS, reactive oxygen species; TAG, triacylglycerol; TG, triglyceride; VSMC, vascular smooth muscle cell.

**Protein**	Atherosclerosis-Related Condition	Perilipin Expression Level	Phenotype	References
Plin1	Plin1 transgenic mice (Plin1 cDNAs under the control of the aP2 enhancer/promoter region) in C57BL/6J background crossed with ApoE^−/−^ mice	Plin1 overexpression	Protection against atheroma progression: reduction in pro-inflammatory macrophages and macrophage polarity change in unstable atheroma plaques	Yamamoto et al., 2018 [59]
Plin2	Plin2^−/−^ mice in C57BL/6J background crossed with ApoE^−/−^ mice	Plin2 inactivation	Protection against atheroma progression: less formation of LDs in foam cells in atherosclerotic lesions	Paul et al., 2008 [60]
Plin2	Human THP-1 monocytes transfected with dicer substrate siRNA to Plin2	Plin2 inactivation	Protection against atherogenesis: increased intracellular TG content, increased LD size, and reduction in pro-atherogenic and pro-inflammatory gene expression	Norman et al., 2018 [61]
Plin2	- Human differentiated THP-1 macrophages incubated with agLDL. - Atherosclerotic foam cells from aortic intimal cells from ApoE^−/−^ mice fed a HFD.	Plin2 overexpression	Stimulation of atherosclerosis development: increase in LD and foam cell formation, autophagy inhibition by chloroquine	Robichaud et al., 2021 [62]
Plin2	Plin2^−/−^ and Plin2^+/+^ C57Bl/6N mice fed with rodent chow diet, under dobutamine-induced stress and myocardial infarction induction	Plin2 overexpression or inactivation	The following was observed in Plin2^−/−^ mice: - Cardiomyocyte-specific increase in lipid storage. - Increased myocardial abundance of Plin3 and Plin5. - Reduction in cardiac function after induced myocardial infarction due to reduced lipophagy.	Mardani et al., 2019 [57]
Plin2 and Plin3	Human differentiated THP-1 macrophages incubated with oxidized low-density lipoproteins	Plin2 overexpression and Plin3 reduction	Stimulation of atherosclerosis development: increase in LD and foam cell formation, significant changes in LD-associated proteins	Li et al., 2010 [63]
Plin3	Human vascular smooth muscle cells cultured with agLDL	Plin3 overexpression	Stimulation of atherosclerosis development and formation of vulnerable atherosclerotic plaques: localization of Plin3 in cellularized regions around the necrotic core	Padro et al., 2017 [64]
Plin3	Murine macrophage RAW264.7 cell line transfected with siRNAs using Lipofectamine2000 and treated with a combination of glucose, insulin, and oleic acid	Plin3 inactivation	Protection against atherogenesis: reduction in LD maturation and TG content in cells but not cholesterol content	Fan et al., 2013 [65] and Gu et al. [66]
Plin5	Plin5^−/−^ mice backcrossed to the C57BL/6J strain	Plin5 inactivation	Decreased LDs in the heart, FA oxidation in cardiomyocytes, increased production of ROS, cardiac dysfunction	Kuramoto et al., 2012 [67]
Plin5	- Plin5-S155A mice (mice with cardiac-specific overexpression of Plin5 encoding a serine-155-to-alanine exchange of the protein kinase A phosphorylation site). - Plin5^+/+^ mice in C57BL/6J background (Plin5 cDNA under the control of the cardiomyocyte-specific MHC promoter).	Plin5 overexpression	Protection from lipotoxicity-induced heart dysfunction: cardiac steatosis and increase in cardiac TAG and ceramide levels, reduced cardiac FA oxidation and lipolysis, reduced mitochondrial fission	Kolleritsch et al., 2020 [68]
Plin5	Plin5^−/−^ mice in C57BL/6J background crossed with ApoE^−/−^ mice	Plin5 inactivation	Stimulation of atherosclerosis development: enhanced inflammation, apoptosis, and oxidative stress	Zhou et al., 2019 [45]
Plin5	Plin5^+/−^ mice in C57BL/6J background	Plin5 inactivation	Stimulation of injury-induced neointima hyperplasia, increased proliferation, and migration of VSMC by inducing oxidative stress	Gan et al., 2022 [69]
